# A validation of the two-high threshold eyewitness identification model by reanalyzing published data

**DOI:** 10.1038/s41598-022-17400-y

**Published:** 2022-08-04

**Authors:** Nicola Marie Menne, Kristina Winter, Raoul Bell, Axel Buchner

**Affiliations:** Department of Experimental Psychology, Heinrich Heine University Düsseldorf, Düsseldorf, Germany

**Keywords:** Psychology, Human behaviour

## Abstract

The two-high threshold (2-HT) eyewitness identification model serves as a new measurement tool to measure the latent cognitive processes underlying eyewitness identification performance. By simultaneously taking into account correct culprit identifications, false innocent-suspect identifications, false filler identifications in culprit-present and culprit-absent lineups as well as correct and false lineup rejections, the model capitalizes on the full range of data categories that are observed when measuring eyewitness identification performance. Thereby, the model is able to shed light on detection-based and non-detection-based processes underlying eyewitness identification performance. Specifically, the model incorporates parameters for the detection of culprit presence and absence, biased selection of the suspect and guessing-based selection among the lineup members. Here, we provide evidence of the validity of each of the four model parameters by applying the model to eight published data sets. The data sets come from studies with experimental manipulations that target one of the underlying processes specified by the model. Manipulations of encoding difficulty, lineup fairness and pre-lineup instructions were sensitively reflected in the parameters reflecting culprit-presence detection, biased selection and guessing-based selection, respectively. Manipulations designed to facilitate the rejection of culprit-absent lineups affected the parameter for culprit-absence detection. The reanalyses of published results thus suggest that the parameters sensitively reflect the manipulations of the processes they were designed to measure, providing support of the validity of the 2-HT eyewitness identification model.

The lineup procedure is an essential tool for assessing eyewitness identifications. In a lineup, a suspect is presented among fillers (known distractors that are not suspected of having committed the crime) to an eyewitness to test the hypothesis that the suspect is the culprit against the hypothesis that the suspect is innocent. Although eyewitness identifications can be a powerful and indispensable form of evidence, the problem of misidentifications has been well documented through DNA-based exonerations^1^. When the lineup includes the culprit (*culprit-present lineup*), the witness may correctly identify the culprit (*correct culprit identification*), but there is also the risk of an incorrect response in that the witness may identify a filler (*false filler identification*) or reject the lineup (*false lineup rejection*). When the suspect is innocent (*culprit-absent lineup*), the witness may either correctly reject the lineup (*correct lineup rejection*) or incorrectly identify the innocent suspect (*false innocent-suspect identification*) or a filler (*false filler identification*). An important goal of eyewitness identification research is to understand the latent cognitive processes underlying these decisions.

When two lineup procedures are compared, the simplest case is that one procedure is clearly superior to the other by yielding both a higher rate of correct culprit identifications in culprit-present lineups and a higher rate of correct lineup rejections of culprit-absent lineups. From such a data pattern, one may conclude that the superior lineup procedure provides better conditions for the process of detecting the culprit’s face in the lineup. However, an increase in the correct culprit identifications is often accompanied by an increase in the false identifications of innocent suspects^2^. This demonstrates that understanding eyewitness identification performance in lineup procedures is complex because the selection of a suspect may not only be caused by the detection of the culprit but also by the biased selection of a suspect who stands out from the fillers, by guessing-based selection among the lineup members or by any combination of these processes. To disentangle the contributions of these different processes to eyewitness identification performance, it is useful to apply a measurement model to the eyewitness identification data. Here, we introduce a novel *two-high threshold (2-HT) eyewitness identification model* for measuring the processes involved in eyewitness identification decisions. The aim of the present article is to test the validity of this model by reanalyzing published data. In an accompanying validation study^3^, we applied the model to novel data that we had collected specifically for the purpose of testing the model’s validity. We consider these two approaches to model validation as complementary: A model should not only be able to account for novel data generated with the model in mind but also for published data of other research groups. To anticipate, both approaches support the validity of the model by showing that the model’s parameters sensitively respond to manipulations of the processes they were designed to measure, suggesting that the model parameters sensitively reflect these processes. Before we describe the 2-HT eyewitness identification model in more detail, we first provide a brief overview of the important discussion about the strengths and weaknesses of different methods for analyzing lineup data that has informed the development of the new measurement tool.

## Measures of eyewitness identification performance in previous research

For some decades, eyewitness identification performance has been measured by using the *diagnosticity ratio* (or other measures of probative value), which is defined as the ratio of the proportion of correct culprit identifications to the proportion of false innocent-suspect identifications^4^. Larger ratios indicate a higher likelihood that an identified suspect is guilty^5^, which may be interpreted to suggest that a lineup procedure that consistently generates a higher diagnosticity ratio should be preferred over one that does not. However, it has been argued that this measure is affected not only by the ability to discriminate culprits from innocent suspects but also by the witness’s response bias, which reflects the overall conservative or liberal tendency to choose someone from the lineup^6–10^. More specifically, the diagnosticity ratio has been shown to increase as a function of an increasingly conservative response bias^9,11^. This discussion has resulted in the application of signal detection theory^12^ to eyewitness identifications. Specifically, Mickes et al.^13^ introduced *Receiver Operating Characteristic* (ROC) analyses to the field of eyewitness identification research.

ROC analyses have the advantage of yielding a measure of discriminability which is not confounded by response bias^13^. In the context of lineups, the term discriminability has been interpreted to refer to a witness’s ability to distinguish culprits from innocent suspects^10,14^. An ROC curve is created by plotting the hit rate (i.e., the proportion of culprit identifications in culprit-present lineups) against the false alarm rate (i.e., the proportion of innocent-suspect identifications in culprit-absent lineups) at different levels of liberal or conservative responding, the latter of which is typically inferred from the witnesses’ post-decision confidence judgements. It has been argued that the lineup procedure associated with the higher ROC curve—indicating higher hit rates and lower false alarm rates—is associated with superior discrimination between the culprit and an innocent suspect and should therefore be preferred^13,15^.

ROC analyses have been developed to account for simple detection tasks with a 2 (signal present, signal absent) × 2 (identification, rejection) data structure (upper half of Table 1). In these tasks, only correct and false identifications are needed for measuring performance because the remaining two data categories (correct and false rejections) are redundant and provide no further information (false rejection rate = 1− correct identification rate; correct rejection rate = 1− false identification rate ^12^). Lineups differ from these simple detection tasks in that they include not only a culprit or an innocent suspect but also fillers. Therefore, in each of the two types of lineups (culprit-present, culprit-absent), witnesses can make one of three responses (suspect identification, filler identification, lineup rejection), resulting in the 2 × 3 data structure displayed in the lower half of Table 1^14,16^. In ROC analyses, filler identifications are treated like lineup rejections and the two data categories are combined to transform the 2 × 3 data structure of lineups into a 2 × 2 data structure. This is justified by noting that filler identifications and lineup rejections have the same legal consequences for the suspect. Irrespective of whether the witness identifies a filler or rejects the lineup, the suspect is not further incriminated by the eyewitness procedure. Therefore, it has been argued that, for the purpose of deciding which of two lineup procedures is superior, it is sufficient to analyze the rate of correct culprit identifications and the rate of false innocent-suspect identifications^13^.

**Table 1:** Comparison of the data structures of the standard signal-detection task and the eyewitness identification task when confronted with a typical lineup.

**2 × 2 data structure of the standard signal-detection task**			
	Identification		Rejection
Signal present	Correct identification		False rejection
Signal absent	False identification		Correct rejection
**2 × 3 data structure of the typical eyewitness identification task**			
	Suspect identification	Filler identification	Rejection
Culprit present	Correct culprit identification	False filler identification	False lineup rejection
Culprit absent	False innocent-suspect identification	False filler identification	Correct lineup rejection

However, if the aim is to understand qualitatively different latent processes underlying eyewitness identification decisions, the two data categories that are combined for ROC analyses can yield important information when analyzed separately. This is so because the underlying processes may differ between identifying a filler and rejecting a lineup. This is already obvious from the fact that the identification of a filler in a culprit-absent lineup is a false response, while the rejection of a culprit-absent lineup is a correct response. In culprit-absent lineups, few correct lineup rejections and many false filler identifications thus indicate poor eyewitness performance, whereas many correct lineup rejections and few false filler identifications indicate good eyewitness performance. Hence, taking into account suspect and filler identifications separately can yield important information about the latent processes underlying eyewitness identification performance^14,16,17^.

## The 2-HT eyewitness identification model

Here, we introduce a new measurement model for eyewitness identification performance that capitalizes on the full range of the data categories observed in typical lineup procedures within *one* model. The model belongs to the class of multinomial processing tree (MPT) models. Models from this class of formal measurement models for categorical data have been successfully applied to different areas within psychology^18,19^ such as memory^20–23^ or decision making^24–26^. Wagenaar and Boer^27^ have already successfully introduced MPT models to the area of eyewitness memory research in a study in which they investigated the processes underlying the misinformation effect. MPT models are based on the assumption that observed response frequencies in a finite set of response categories can arise from combinations of latent processes that can be depicted in a tree-like structure^28^. Each cognitive process is represented by a model parameter that serves to measure the probability with which the process occurs^19,29^. Parameter estimation is achieved by employing the expectation-maximization algorithm proposed by Hu and Batchelder^30^. The algorithm aims to determine a set of parameters that minimize the distance between the observed response frequencies and the response frequencies predicted by the model, as measured by the log-likelihood ratio goodness-of-fit statistic *G*^2^^30–32^. If the deviation between the frequencies predicted by the model and the observed response frequencies is not statistically significant, it can be assumed that the model fits the data^19,29^. A model is called identifiable when a unique set of parameter estimates provides an optimal fit for a given set of observed response frequencies^19,29^. Under these circumstances, MPT models can be used to test hypotheses directly at the level of the model parameters. Hypotheses are tested by imposing theoretically motivated restrictions on model parameters. If the restricted model provides a significantly worse fit to the data than the model without the restriction (measured by the ∆*G*^2^ difference statistic), then the assumption implied by the restriction has to be rejected^18,19,29^. Thereby, this method provides insights into the latent processes underlying observable behavior, which is a major advantage of MPT models given that psychological theories often involve hypotheses about cognitive processes. Parameter estimation and statistical tests can be performed with freely available computer programs^28,32,33^.

**Figure 1:**
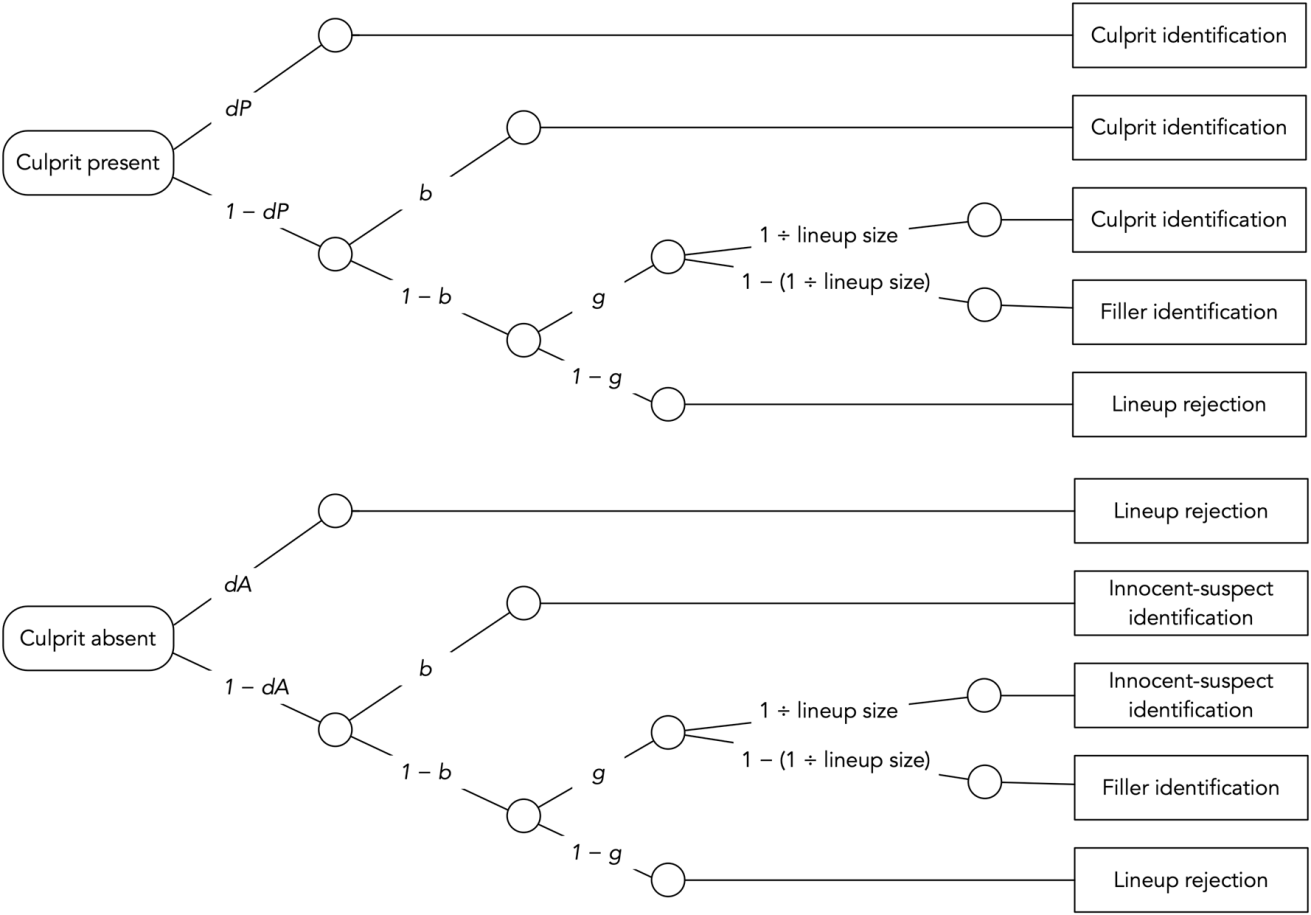
Graphical illustration of the 2-HT eyewitness identification model. Rounded rectangles on the left represent the two types of lineups presented to the participants (culprit-present and culprit-absent). Rectangles on the right represent the observable response categories. The letters attached to the branches represent the probabilities of the latent cognitive processes postulated by the model (*dP* = probability of detecting the presence of the culprit; *b* = probability of biased selection of the suspect; *g* = probability of guessing-based selection among the lineup members; lineup size = the number of persons in the lineup; *dA* = probability of detecting the absence of the culprit).

The 2-HT eyewitness identification model is illustrated in Fig. 1. The structure of the model is congenial to that of MPT models designed to measure the processes underlying performance in other recognition paradigms [e.g.,^34,35^]. The model comprises two trees, one for each of the two possible types of lineups presented to the witnesses (culprit-present and culprit-absent lineups). If the culprit is present (upper tree in Fig. 1), the presence of the culprit may be detected with probability *dP* (for *detection of the presence of the culprit*), which results in the correct identification of the culprit. The detection state is based on the witnesses’ memory of the culprit. If witnesses fail to detect the culprit, which occurs with the complementary probability 1 − *dP*, then two different types of non-detection-based processes may still lead to correct culprit identifications. First, the lineup may be unfair in that the suspect stands out from the fillers so that it can be inferred who the suspect is without relying on memory. One telling example is the case of Marvin Lamont Anderson who served fifteen years in prison for a rape that he did not commit. The selection of Anderson’s face in the police lineup was most likely due to the fact that the lineup was unfair: The witness was shown a color identification card of Anderson along with six black-and-white mug shots that served as fillers^36^. The false identification of Anderson as the culprit was not based on a culprit-detection process but most likely due to the biased selection of the color photo of Anderson that stood out from the black-and-white photos of the fillers. The process of *biased selection* of the suspect in unfair lineups is reflected in parameter *b*. In culprit-present lineups, the biased selection of the suspect yields a correct identification of the culprit. Second, if a biased selection of the suspect does not occur (with probability 1 − *b*), then witnesses may still select one of the lineup members based on guessing with probability *g* (for *guessing-based selection*). Guessing-based selection leads to the identification of the culprit with a probability that is equal to 1 ÷ lineup size. For instance, in a lineup with six persons, the probability that guessing-based selection will lead to the identification of the culprit is 1/6. With the complementary probability (e.g., 5/6 in a six-person lineup), the witnesses will identify a filler. Alternatively (with probability 1 − *g*), the witnesses may abstain from selecting a lineup member based on guessing, which leads them to incorrectly reject the culprit-present lineup.

The lower tree illustrates the cognitive processes in response to lineups in which the culprit is absent. Witnesses may correctly detect that the culprit is absent and that no other person in the lineup can possibly be the culprit with probability *dA* (for *detection of the absence of the culprit*), which results in the correct rejection of the lineup. This may occur, for instance, if all persons in the lineup have a birthmark and a witness then remembers that the culprit did not have a birthmark. When the absence of the culprit is not detected (with probability 1 − *dA*), the witnesses rely on the same non-detection-based biased and guessing-based processes as in culprit-present lineups. With probability *b*, biased selection may occur if the innocent suspect stands out from the fillers in an unfair lineup. Biased selection leads to the false identification of the innocent suspect. If no biased selection occurs (with probability 1 − *b*), the witnesses may select one of the lineup members based on guessing with probability *g*. The probability with which guessing-based selection leads to the identification of the innocent suspect or one of the fillers depends on the lineup size (in a six-person lineup, the probability of identifying the innocent suspect is 1/6 and the probability of choosing one of the fillers is 5/6). If the witnesses abstain from selecting a lineup member based on guessing (with probability 1 − *g*), the culprit-absent lineup is correctly rejected.

The 2-HT eyewitness identification model belongs to the class of 2-HT recognition models [cf.^35,37–39^]. Parameter *dP* indicates the probability of crossing the threshold for culprit-presence detection, whereas *dA* indicates the probability of crossing the threshold for detecting the absence of the culprit. To make 2-HT models in standard recognition tests identifiable, it is often assumed that the probability of detecting the presence of a signal is equal to the probability of detecting the absence of the signal [e.g.,^37^]. Here, we have the advantage that both detection parameters (*dP* and *dA*) can vary freely due to the increased number of independent observable data categories in lineups compared to simple old-new recognition tests [cf.^40^]. The 2-HT eyewitness identification model can be transformed into a one-high threshold model by setting parameter *dA* to zero^41^. The fact that *dA* does not have to be set to a certain value to obtain model identifiability implies that it is possible to empirically test whether witnesses are able to detect the absence of a culprit or not. To anticipate, the results of novel validation studies^3^ and the present reanalyses of existing data consistently indicate that witnesses do not always spontaneously succeed in detecting the absence of the culprit but that there are circumstances in which witnesses can in fact detect the absence of the culprit (see the section on the validation of parameter *dA* below). When no further restrictions are employed, then the 2-HT eyewitness identification model has zero degrees of freedom, implying that there are as many free parameters as there are independent data categories to fit.

Importantly, the parameters of a novel MPT model need to be validated^19,29^. A crucial first step of model validation entails testing whether the parameters of the model measure the processes they were designed to measure. This is achieved by testing, for each model parameter, whether a parameter intended to represent a specific cognitive process is affected by experimental manipulations that target this process^18^. We decided to take two complementary approaches to model validation. In one approach^3^, novel data were collected to target the model parameters one by one. Here, we test whether the parameters of the 2-HT eyewitness identification model are valid given published data from other research groups. We see these approaches as complementary. The following criteria were employed to select the data sets for the present reanalyses: First, the data had to be reported in sufficient detail, which is not the case for a surprisingly large number of studies. Second, the effect of the experimental manipulation on a model parameter had to be as obvious as possible a priori. In validation experiments, manipulations are needed for which there is a straightforward relationship between the factors manipulated and the cognitive processes that can be expected to be influenced by those manipulations. Third, the study design had to be of minimal complexity to keep the reanalysis as simple as possible. For each of the four parameters of the 2-HT eyewitness identification model, the first two studies that fulfilled these requirements were analyzed. First, we validated the culprit-presence detection parameter *dP* by testing whether the duration of the exposure to the culprit’s face and the viewing conditions at encoding affect the participants’ ability to detect the culprit’s presence in the lineup. Second, we validated the biased-suspect-selection parameter *b* by reanalyzing the data of two studies in which lineup fairness was manipulated. Third, we tested whether the guessing-based selection parameter *g* differs between one-sided and two-sided pre-lineup instructions. One-sided instructions insinuate that the culprit is in the lineup, whereas two-sided instructions emphasize that the culprit might or might not be in the lineup. Finally, we completed the model validation by showing that techniques developed to help children and older adults to reject culprit-absent lineups selectively affect the culprit-absence detection parameter *dA*.

## Manipulations of culprit-presence detection: Validation of parameter *dP*

Our first aim was to validate parameter *dP*, which represents the probability of detecting the presence of the culprit. The probability of detecting the culprit’s face can be expected to increase with the duration of exposure to the culprit’s face based on the results of recognition [for a meta-analysis, see^42^] and staged-crime studies^43,44^ showing better detection performance following longer exposure. Here, we tested whether these effects of exposure duration are sensitively reflected in parameter *dP* by reanalyzing the data of Memon et al.^45^ who had manipulated culprit-exposure duration at encoding. We also applied the 2-HT eyewitness identification model to the data of Smith [^46^, Experiment 1] who had manipulated the viewing conditions at encoding. When the viewing conditions at encoding are poor, the culprit’s face should provide a weaker match to memory, which should hinder culprit-presence detection.

### Effects of exposure time on culprit-presence detection: Reanalysis of Memon et al.^45^

In the study of Memon et al.^45^, participants viewed a simulated staged-crime video in which they saw the culprit’s face for either a long or a short duration. In line with previous research^43,44^, Memon et al. found that participants were better able to identify the culprit under the long than under the short exposure duration. The model-based reanalysis of these data should show that the manipulation of exposure duration affects parameter *dP.* More precisely, parameter *dP* should be significantly higher for the long compared to the short exposure condition.

#### Method

Memon et al.^45^ randomly assigned younger (age: 17 to 25 years, *n* = 84) and older (age: 59 to 81 years, *n* = 80) participants to one of the four conditions resulting from a 2 (exposure duration: short vs. long) × 2 (culprit presence: present vs. absent) between-subjects design. Participants saw a staged-crime video depicting a robbery. The video was long (100 s) or short (67 s). The long video involved a clear exposure to the face of the main culprit for 45 s. The short video provided a full-face and profile-view exposure to the face of the main culprit for only 12 s. After having completed several filler questionnaires, participants were given standard two-sided pre-lineup instructions. Specifically, participants were informed that the culprit may or may not be in the lineup. Participants were then asked to identify one of the lineup members as the culprit or to indicate that the culprit was absent. Half of the participants saw a culprit-present black-and-white photo lineup consisting of six faces in a 3 × 2 array. For the remaining half of the participants, the culprit was replaced by another filler to construct a culprit-absent lineup. All fillers matched the culprit’s general description. Both the culprit and the filler that replaced the culprit were positioned in the top right-hand corner of the array [for more details, see^45^].

#### Results

**Figure 2:**
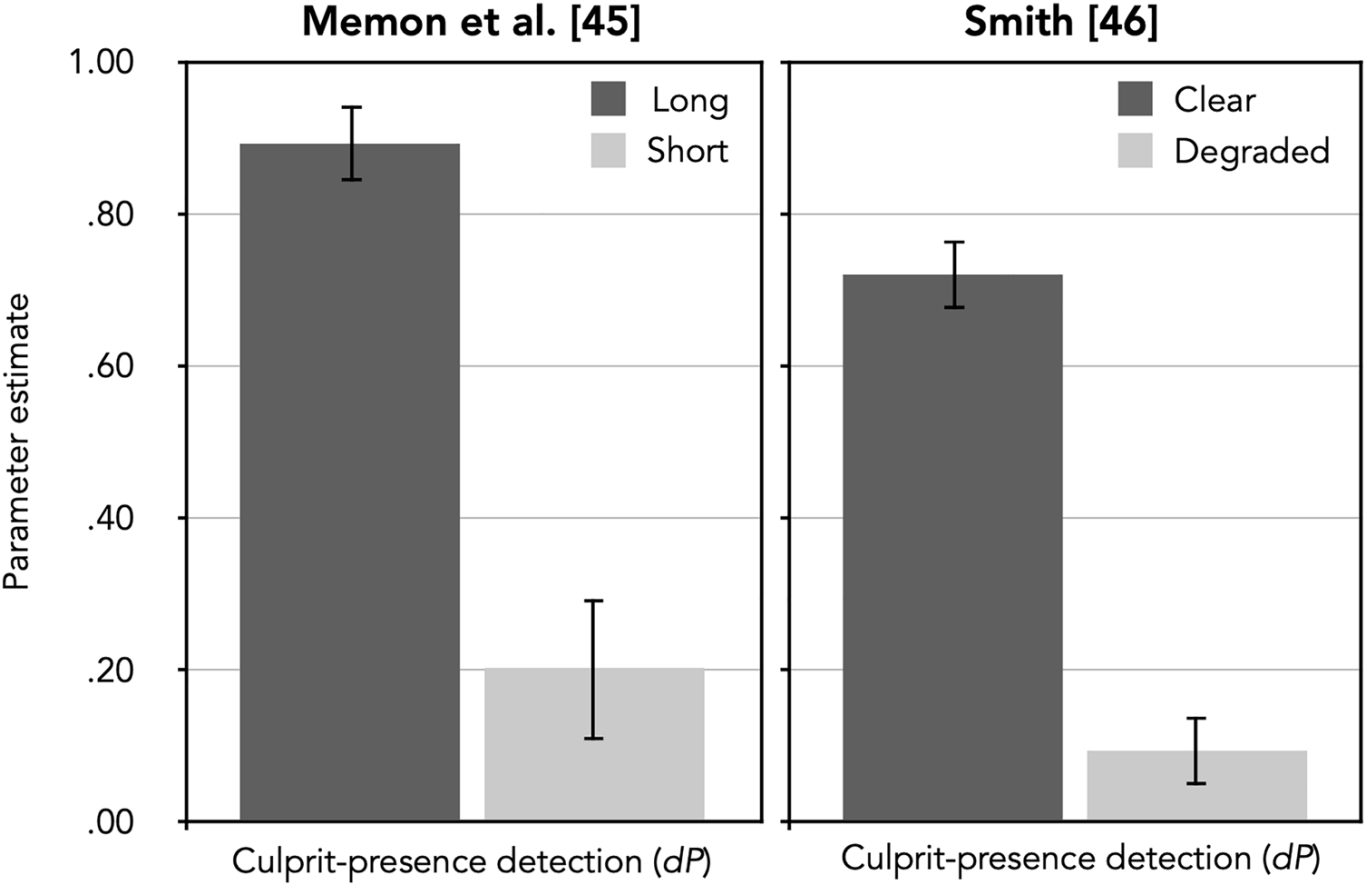
Estimates of parameter *dP* (representing the probability of detecting the presence of the culprit) of the 2-HT eyewitness identification model when applied to the data reported by Memon et al.^45^ and by Smith [^46^, Experiment 1] as a function of exposure duration (long vs. short; left panel) and viewing conditions at encoding (clear vs. degraded; right panel). The error bars represent standard errors.

For all analyses reported in this article, parameter estimates were obtained and likelihood-ratio tests were performed using *multiTree*^32^. The *α* level was set to 0.05. The observed response frequencies (see the upper half of Table 2) were reconstructed from the data reported in Memon et al.’s^45^ Table 1 in which the exact number of participants for each condition is not provided. We therefore divided the total number of participants in each age group by four (the number of conditions in each age group) to estimate the number of participants in each condition. Following standard practice [cf.^47,48^], the number of innocent-suspect identifications was estimated by dividing the total number of false identifications in culprit-absent lineups by the number of lineup members (in this case, six). Likewise, the number of filler identifications in culprit-absent lineups was estimated by subtracting the number of innocent-suspect identifications from the total number of false identifications. To test how the manipulation of exposure duration affects parameter *dP*, four sets of the processing trees displayed in Fig. 1 were needed, one for the long and one for the short exposure duration, for both the younger and the older participants, respectively. Parameters *dA* and *b* were each set to be equal among the four conditions because there was no reason to assume that these parameters should differ as a function of the conditions. To simplify the output, we also aggregated the data for younger and older participants by imposing the equality restrictions that culprit-presence detection (*dP*) and guessing-based selection (*g*) did not differ between the two age groups in each exposure condition. These assumptions were based on the results of Memon et al. who had found no significant effect of age on lineup performance. The model incorporating these restrictions was used as a comparison standard for the subsequent nested likelihood-ratio tests, *G*^*2*^(10) = 8.19, *p* = 0.610. The estimates of parameter *dP* as a function of exposure duration are shown in the left panel of Fig. 2. Table 3 shows the estimates of parameters *b*, *g* and *dA*.

**Table 2:** Response frequencies as reconstructed from Table 1 of Memon et al. [^45^, p. 345] and from Table 1 of Smith [^46^, p. 500], see text for details.

	Culprit-present lineups			Culprit-absent lineups		
	Culprit identifications	Filler identifications	Lineup rejections	Innocent-suspect identifications	Filler identifications	Lineup rejections
**Memon et al.**^45^						
Exposure duration						
Younger participants						
Long	20	1	0	2	7	12
Short	6	9	6	3	16	2
Older participants						
Long	17	2	1	2	8	10
Short	7	9	4	3	13	4
**Smith**^46^						
Viewing conditions						
Clear	79	9	20	9	43	153
Degraded	17	35	47	18	89	96

**Table 3:** Estimates of parameters *b*, *g* and *dA* of the 2-HT eyewitness identification model for the data reported by Memon et al.^45^ and by Smith [^46^, Experiment 1].

Memon et al.^45^				Smith^46^			
Exposure duration	Parameter estimates			Viewing conditions	Parameter estimates		
	*b*	*g*	*dA*		*b*	*g*	*dA*
Long	.02 (.04)	.48 (.09)	.00 (.12)	Clear	.00 (.01)	.26 (.04)	.00 (.12)
Short		.78 (.06)		Degraded		.51 (.05)	

Values in parentheses represent standard errors. Within the model used as a comparison standard, parameter *g* was estimated separately for long and short exposure conditions^45^ and for clear and degraded viewing conditions [^46^

As explicated above, the 2-HT eyewitness identification model is based on the full 2 × 3 structure of lineup data. To use the model for the present reanalysis and that of all other data sets without a designated innocent suspect, it is thus necessary to make the assumption that the culprit-absent lineups contained an innocent suspect and that the lineups were fair, which implies that the innocent suspects were selected with the same probability as each of the fillers. Given that the proportion of innocent-suspect identifications is determined from the proportion of filler identifications, one could argue that, technically speaking, experiments without a designated innocent suspect have one fewer independent data category than experiments with a designated innocent suspect. As noted by a reviewer during the review process, this would result in a loss of degrees of freedom in the model used as a comparison standard. However, the same applies to all nested models generated by parameter restrictions (see below). Therefore, none of the subsequent likelihood-ratio tests, all of which pertain to the *differences* between the models used as comparison standards and the more restricted, nested models that are central to the model validation are affected by this issue.

The culprit-presence detection parameter *dP* was clearly affected by exposure duration. Participants in the long-exposure group were significantly more likely to detect the culprit than those in the short-exposure group, ∆*G*^2^(1) = 34.33, *p* < 0.001. Furthermore, the probability of selecting a lineup member based on guessing (parameter *g*) was significantly higher when exposure duration was short than when it was long, ∆*G*^2^(1) = 10.24, *p* = 0.001, likely reflecting the well-established phenomenon of compensatory guessing, that is, the phenomenon that participants tend to rely more on guessing when memory is poor^23,49–54^*.*

### Effects of viewing conditions on culprit-presence detection: Reanalysis of Smith^46^

Smith [^46^, Experiment 1] manipulated the viewing conditions at encoding by presenting participants with either a clear or a degraded version of a simulated-crime video. In line with the results of Smith et al. [^55^, Experiment 1], degraded viewing conditions decreased culprit detection performance. If the 2-HT eyewitness identification model is valid, the manipulation of viewing conditions should affect the culprit-presence detection parameter *dP*. Specifically, parameter *dP* should be significantly higher for the clear compared to the degraded viewing conditions.

#### Method

Smith [^46^, Experiment 1] randomly assigned 615 participants to one of the four experimental conditions resulting from a 2 (viewing conditions: clear vs. degraded) × 2 (culprit presence: present vs. absent) between-subjects design. Half of the participants viewed the video of the culprit in high-resolution so that the culprit’s facial features were clearly visible. The remaining participants saw a low-resolution, overexposed version of the same video in which it was extremely difficult to perceive facial details. In both versions of the 90-s video, a scene at an airport was shown in which the culprit switched his suitcase with that of another person. After having completed a 4-min anagram task, participants were given standard two-sided pre-lineup instructions and were then presented with a simultaneous lineup. The data from the culprit-absent lineups were crucial for testing Smith’s hypothesis. Therefore, approximately two-thirds of the participants were presented with the culprit-absent lineup containing six fillers. About one-third of the participants saw a six-person culprit-present lineup that included the culprit together with five of the six fillers who were randomly selected. All fillers matched the culprit’s general description. The lineup members were presented in random order [for more details, see^46^, Experiment 1].

#### Results

The observed response frequencies (see the lower half of Table 2) of lineup identifications and rejections were taken from Table 1 in Smith^46^. As in the previous analysis, we estimated the number of innocent-suspect identifications using the standard procedure of dividing the total number of false identifications in culprit-absent lineups by the numb er of lineup members [cf.^47,48^]. For the model-based reanalysis, we needed two sets of the processing trees depicted in Fig. 1, one for the clear and one for the degraded viewing conditions. As in the previous analysis, parameters *dA* and *b* were each set to be equal between both conditions because there was no reason to assume that these parameters should differ as a function of the viewing conditions. The model incorporating these restrictions was used as a comparison standard for the subsequent nested likelihood-ratio tests, *G*^2^(2) = 1.81, *p* = 0.405. The estimates of culprit-presence detection parameter *dP* as a function of viewing conditions are shown in the right panel of Fig. 2. Table 3 shows the estimates of parameters *b*, *g* and *dA*.

The model-based reanalysis confirmed that parameter *dP* sensitively reflected the effect of the viewing conditions at encoding. The probability of correctly detecting the presence of the culprit was significantly higher under clear than under degraded viewing conditions, ∆*G*^2^(1) = 74.73, *p* < 0.001. In addition, parameter *g* was significantly higher when viewing conditions were poor, ∆*G*^2^(1) = 32.02, *p* < 0.001, which can be attributed to compensatory guessing^23,49–54^.

### Discussion

In reanalyzing data obtained from the literature^45,46^, we first focused on parameter *dP* of the 2-HT eyewitness identification model. Parameter *dP* reflects the detection of the presence of the culprit in a lineup. Both exposure duration and viewing conditions can be predicted clearly and unambiguously to affect the ability to detect the culprit in culprit-present lineups. Parameter *dP* sensitively reflected the two different manipulations of culprit-presence detection. The results of the first reanalysis^45^ confirmed that parameter *dP* was significantly higher under long than short exposure to the culprit at encoding. The results of the second reanalysis [^46^, Experiment 1] demonstrated that the culprit-presence detection parameter *dP* was significantly higher when viewing conditions at encoding were good than when they were poor. It can thus be concluded that parameter *dP* sensitively reflects manipulations of culprit-presence detection in the predicted directions, which is consistent with the theoretical interpretation of the results in the original studies^45,46^. This suggests that the validation of parameter *dP* was successful.

In both reanalyses, the guessing-based selection parameter *g* was also affected by the manipulations of encoding difficulty. Participants were more likely to select one of the lineup members based on guessing when culprit-presence detection was poor. Ideally, the procedure of model validation entails experimental manipulations that only influence the target parameter (*dP* in this instance) in the expected direction without affecting other parameters. However, it is often not possible to find strong manipulations that influence only a single cognitive process without side effects on other processes^29^. In such a case it is ideal if there is a plausible explanation for these side effects. In the present case, the side effect on the guessing-based selection parameter *g* can be explained by compensatory guessing, which refers to the well-established phenomenon that participants rely more on guessing when memory is poor^23,49–54^. The effect of compensatory guessing may thus be linked to the fact that the differences in memory between the conditions was rather strong in the reanalyzed studies of Memon et al.^45^ and of Smith^46^.

## Manipulations of biased suspect selection: Validation of parameter *b*

Our second objective was to investigate the validity of parameter *b*, which serves to measure the probability of biased selection of the suspect from a lineup if the suspect stands out from the other lineup members. Thus, the estimate of *b* should primarily be determined by the degree of lineup fairness. Specifically, parameter *b* should be higher for unfair lineups than for fair lineups. If a lineup is perfectly fair, there is no way to tell the suspect apart from the other lineup members without memory of the culprit. Under these conditions, biased selection of the suspect should not be possible and parameter *b* should be indistinguishable from zero. Note that this is what happened in the reanalyses of Memon et al.^45^ and Smith^46^ in which we followed the standard procedure of dividing the total number of false identifications in culprit-absent lineups by the number of lineup members [cf.^47,48^] to obtain an estimate of the innocent-suspect identifications in culprit-absent lineups. However, it is well known that real-world lineups are often not perfectly fair and there is strong evidence of a biased selection of suspects in unfair lineups [e.g.,^56–58^]. If the 2-HT eyewitness identification model is valid, then the biased selection of suspects in unfair lineups should be sensitively reflected in parameter *b*. This was tested by reanalyzing data from two large studies ^48,59^ in which lineup fairness was manipulated. Specifically, Wetmore et al.^59^ observed an effect on lineup fairness when manipulating the similarity between the suspect and the fillers. The study of Colloff et al.^48^ provides a complementary approach to manipulating the biased selection of suspects by eliminating, via digital photo manipulation, distinctive features that caused the suspect to stand out from the fillers.

### Effects of filler-suspect similarity on biased suspect selection: Reanalysis of Wetmore et al.^59^

Wetmore et al.^59^ manipulated the degree of similarity between the lineup fillers and the suspect. Good fillers (high similarity to the suspect) were used to create a fair lineup, whereas bad fillers (low similarity to the suspect) were used to create an unfair lineup. Wetmore et al. found significantly more suspect identifications when the lineup was unfair than when it was fair. The theoretical interpretation of this finding was that the higher rate of suspect identifications in unfair lineups was due to biased suspect selection. Therefore, Wetmore et al.’s lineup fairness manipulation should be reflected in the 2-HT eyewitness identification model’s parameter *b*. Specifically, parameter *b* should be significantly larger for the unfair lineup than for the fair lineup if the interpretation of parameter *b* in terms of biased suspect selection is valid.

#### Method

Wetmore et al.^59^ randomly assigned 1584 participants to one of the 18 conditions of a 3 (identification task: showup vs. fair lineup vs. unfair lineup) × 3 (suspect: guilty vs. Innocent 1 vs. Innocent 2) × 2 (delay: immediate vs. delayed) between-subjects design. Participants watched a 105-s video of a man stealing a woman’s purse. Participants completed a distractor task that consisted of solving 20 anagrams either immediately or 48 h after the simulated crime had been shown. Standard two-sided pre-lineup instructions were given before the participants proceeded to the identification task. Participants in the lineup condition viewed a six-person simultaneous lineup composed of two rows of three photos. The lineups included the culprit (culprit-present lineup) or an innocent suspect (culprit-absent lineup) who matched the culprit’s description. Wetmore et al. distinguished between two innocent suspects that had been taken from an earlier study by Gronlund et al.^60^. The innocent suspects were intended to be equally good matches to the culprit. For simplicity, we did not distinguish between the two innocent suspects. The participants saw either a fair or an unfair lineup*.* To create unfair lineups in which the suspect stood out, five poor fillers were selected who, apart from being white men, shared only one characteristic with the culprit. In contrast, fair lineups contained good fillers who, apart from being white men, shared five characteristics with the culprit [for more details, see^59^].

#### Results

**Figure 3:**
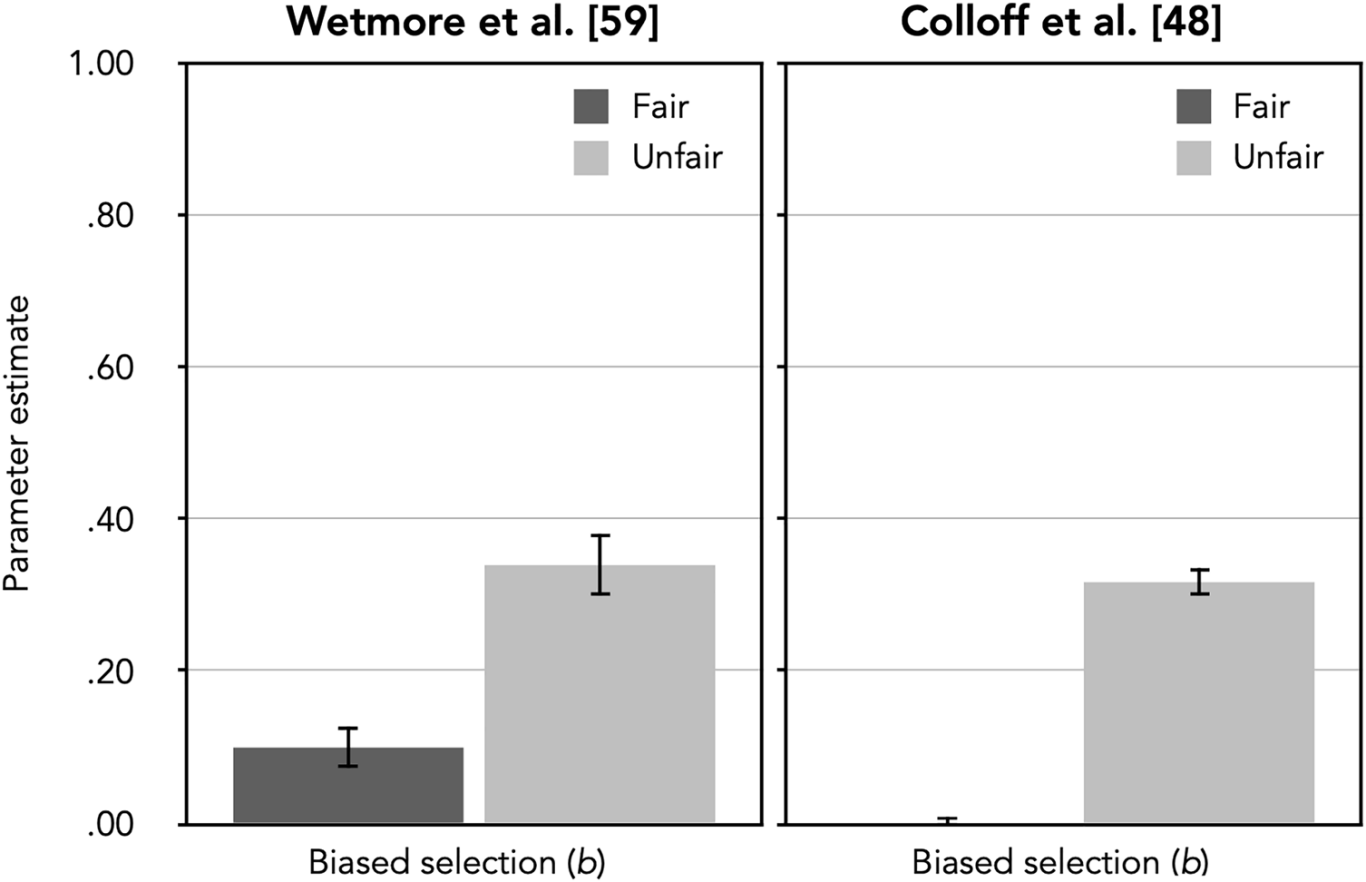
Estimates of parameter *b* (representing biased selection of the suspect) of the 2-HT eyewitness identification model when applied to the data reported by Wetmore et al.^59^ and by Colloff et al.^48^ as a function of lineup fairness (fair vs. unfair). The error bars represent standard errors.

The observed response frequencies (see the upper half of Table 4) were reconstructed from the proportions presented by Wetmore et al.^59^ in their Table 2. For simplicity, we limited our reanalysis to the lineup data to focus on the most relevant comparison (fair vs. unfair) for testing the validity of parameter *b.* Thus, four sets of the trees shown in Fig. 1 were needed, one for immediate fair, one for immediate unfair, one for delayed fair and another for delayed unfair lineups. The *dA* parameters were set to be equal among the four conditions because there was no reason to assume that the detection of culprit absence should differ as a function of the conditions. Given that Wetmore et al. had found no effect of delay on identification performance, we reduced the model complexity by assuming that biased suspect selection (*b*), culprit-presence detection (*dP*) and guessing-based selection (*g*) did not differ between immediate and delayed lineups. The model incorporating these restrictions was used as a comparison standard for the subsequent nested likelihood-ratio tests, *G*^2^(9) = 14.10, *p* = 0.119. The estimates of the biased-suspect-selection parameter *b* as a function of lineup fairness are shown in the left panel of Fig. 3. Table 5 shows the estimates of parameters *dP*, *g* and *dA*.

**Table 4:** Response frequencies as reconstructed from Table 2 of Wetmore et al. [^59^, p. 11] and from Table 2 of Colloff et al. [^48^, p. 1235], see text for details.

	Culprit-present lineups			Culprit-absent lineups		
	Culprit identifications	Filler identifications	Lineup rejections	Innocent-suspect identifications	Filler identifications	Lineup rejections
**Wetmore et al.**^59^						
Lineup fairness						
Immediate						
Fair	41	6	13	31	74	54
Unfair	57	2	11	56	37	58
Delay						
Fair	59	13	14	22	59	38
Unfair	54	4	14	48	31	39
**Colloff et al.**^48^						
Lineup type						
Block	323	390	414	101	503	534
Pixelation	320	411	414	102	512	510
Replication	347	382	396	105	523	513
Unfair	629	206	275	364	219	434

**Table 5:** Estimates of parameters *dP, g* and *dA* of the 2-HT eyewitness identification model for the data reported by Wetmore et al.^59^ and by Colloff et al.^48^.

Wetmore et al.^59^				Colloff et al.^48^			
Lineup fairness	Parameter estimates			Lineup fairness	Parameter estimates		
	*dP*	*g*	*dA*		*dP*	*g*	*dA*
Fair	.61 (.05)	.61 (.05)	.00 (.07)	Fair	.22 (.01)	.55 (.01)	.02 (.02)
Unfair	.64 (.06)	.42 (.05)		Unfair	.32 (.03)	.43 (.02)	

Values in parentheses represent standard errors. Within the model used as a comparison standard, parameters *dP* and *g* were estimated separately for fair and unfair lineups. Parameter *dA* was set to be equal between the fair and the unfair-lineup conditions. *dP* = probability of detecting the presence of the culprit; *g* = probability of guessing-based selection among the lineup members; *dA* = probability of detecting the absence of the culprit.

The fairness manipulation affected parameter *b* as predicted under the assumption that this parameter represents biased suspect selection. The probability of biased suspect selection was significantly higher in the unfair than in the fair-lineup condition, ∆*G*^2^(1) = 31.84, *p* < 0.001. In addition, guessing-based selection was more prevalent when the fillers matched the description of the culprit than when they did not match the description: Parameter *g* was decreased in the unfair in comparison to the fair-lineup condition, ∆*G*^2^(1) = 15.42, *p* < 0.001. This is to be expected because guessing-based selection among the lineup members should have been discouraged in the unfair lineup due to the poor match of the fillers to the description of the culprit. Parameter *dP*, which measures the ability to detect the presence of the culprit, was not affected by lineup fairness, ∆*G*^2^(1) = 0.19, *p* = 0.665.

### Effects of the elimination of distinctive features of the suspect on biased suspect selection: Reanalysis of Colloff et al.^48^

Colloff et al.^48^ presented participants with staged-crime videos in which the culprits had distinctive facial features. To create fair lineups, Colloff et al. tested three possible techniques to prevent distinctive suspects from standing out: blocking, pixelating or replicating the feature on all faces in the lineup using digital photo-manipulation technology. Colloff et al. also included an unfair lineup in which only the suspect had the distinctive feature. The results showed that if a suspect stood out, identifications shifted from the fillers to the suspect. Thus, unfair lineups led to significantly more suspect identifications than fair lineups. If the 2-HT eyewitness identification model is valid, the biased-suspect-selection parameter *b* should be affected by the manipulation of fairness. Specifically, parameter *b* should be higher for the unfair than for the fair lineups.

#### Method

Colloff et al.^48^ randomly assigned 8925 participants to one of the eight conditions of a 4 (lineup type: block vs. pixelation vs. replication vs. unfair) × 2 (culprit presence: present vs. absent) between-subjects design. The participants viewed one of four 30-s crime videos (carjacking, graffiti attack, mugging, theft) depicting four different culprits, each with a unique and distinctive facial feature (scar on the left cheek, bruising around the right eye, nose piercings in the left nostril or facial tattoo on the right cheek). After an 8-min retention interval, participants were presented with a simultaneous six-person lineup composed of two rows of three photos. The lineup consisted of either one culprit and five fillers (culprit-present lineup) or six fillers (culprit-absent lineup). The fillers were randomly drawn from a pool of 40 description-matched fillers created for each culprit. Depending on the condition, the distinctive feature of the culprit was treated differently. Three treatments were intended to produce fair lineups by (a) concealing the distinctive feature with a solid black rectangle on the culprit and by covering the equivalent area on each filler (block), (b) pixelating the distinctive feature on the culprit and the equivalent area on each filler (pixelation) or (c) digitally adding the distinctive feature to each filler (replication). In the unfair condition, participants saw a lineup in which the suspect stood out due to them being the only person with a distinctive feature. For unfair culprit-present lineups, Colloff et al. left the distinctive feature on the culprit unaltered. For each unfair culprit-absent lineup, a replication filler photo with the culprit’s distinctive feature was randomly selected to create an innocent suspect with the culprit’s distinctive feature while the other five filler photos remained unedited. For all lineups, the position of the culprit (in culprit-present lineups) and innocent suspect (in unfair culprit-absent lineups) was randomly determined. Before viewing the lineup, standard two-sided pre-lineup instructions were presented [for more details, see^48^].

#### Results

The observed response frequencies (see the lower half of Table 4) were provided by Colloff et al.^48^ in their Table  2 (rounded to the next integer, where applicable). Four sets of the model trees depicted in Fig. 1 were necessary to reanalyze the data, three for the fair-lineup conditions (block, pixelation, replication) and one for the unfair condition. The *dA* parameters were set to be equal among the four conditions because there was no reason to assume that the detection of culprit absence should differ as a function of the conditions. In Colloff et al., the three fair-lineup conditions were all associated with similar levels of performance. To keep the model as simple as possible, we therefore assumed that biased suspect selection (*b*), culprit-presence detection (*dP*) and guessing-based selection (*g*) did not differ among the three fair-lineup conditions. The model incorporating these restrictions was used as a comparison standard for the subsequent nested likelihood-ratio tests, *G*^2^(9) = 12.92, *p* = 0.166. The estimates of the biased-suspect-selection parameter *b* as a function of lineup fairness are shown in the right panel of Fig. 3. Table 5 shows the estimates of parameters *dP*, *g* and *dA*.

Parameter *b* adequately reflected the fairness manipulation. The probability of biased suspect selection was significantly higher in the unfair-lineup condition compared to the fair-lineup condition, ∆*G*^2^(1) = 418.89, *p* < 0.001. In addition, parameter *g*, which represents the probability of selecting one of the lineup members based on guessing, was significantly decreased in the unfair-lineup condition in comparison to the fair-lineup condition, ∆*G*^*2*^(1) = 48.99, *p* < 0.001. This is to be expected given that guessing-based selection among the lineup members was discouraged by the fact that none of the fillers shared the culprit’s distinctive facial feature. This is parallel to what was observed in the data of Wetmore et al.^59^ considered in the previous section. The ability to detect the presence of the culprit, captured by parameter *dP,* was significantly higher in unfair than in fair lineups, ∆*G*^2^(1) = 8.37, *p* = 0.004, which can be explained by attention being drawn to faces with the culprit’s distinctive facial features.

### Discussion

The results support the validity of parameter *b* representing the process of biased suspect selection. In the two experiments that were reanalyzed, unfair lineups were created either by using fillers with low similarity to the suspect^59^ or by using a suspect with an uncovered distinctive facial feature that makes the suspect stand out from the fillers^48^. Both model-based reanalyses demonstrated parameter *b* to be significantly higher for unfair lineups than for fair lineups. Parameter *b* thus sensitively reflects manipulations of biased suspect selection.

When applying the 2-HT eyewitness identification model to Colloff et al.’s^48^ data, the culprit-presence detection parameter *dP* was also affected by lineup fairness. Better detection of culprit presence in unfair lineups can be easily explained by distinctive facial features drawing attention to the culprit’s face. This finding is in line with the well-established principle that the presence of highly similar distractors at test decreases memory performance [cf.^14,61,62^]. In both reanalyses, parameter *dP* was higher in unfair than in fair lineups, but this difference reached significance only in the reanalysis of the Colloff et al. data. Given that sample size was much larger in the study of Colloff et al. than in the study of Wetmore et al.^59^, this discrepancy is expected if the secondary effect of lineup fairness on culprit-presence detection is more subtle than the primary effect of lineup fairness on biased selection and can thus only be detected when the sample size provides the statistical power to detect a subtle effect. At first glance, better culprit detection in unfair lineups may seem unexpected given that unfair lineups were associated with an impaired ability to discriminate between culprits and innocent suspects in the analyses of Colloff et al. and Wetmore et al. However, their conclusions relied on ROC analyses and thus only on the correct culprit identifications and false innocent-suspect identifications. As Smith et al.^62^ have already pointed out, the finding of increased culprit-presence detection in Colloff et al.’s unfair lineups is already apparent at the surface level of the raw response frequencies when considering the full 3 × 2 matrix of lineup data. The effect of the fairness manipulation on biased selection is obvious when looking at the false responses: Participants chose the innocent suspect in culprit-absent lineups more often than a filler when the lineup was unfair but chose one of the fillers more often than the innocent suspect when the lineup was fair. In the 2-HT eyewitness identification model, this effect is reflected in the biased-suspect-selection parameter *b*. However, when looking at the correct responses, it is clear that culprit-presence detection was better in unfair lineups than in fair lineups: In culprit-present lineups, participants made more correct identifications when the lineups were unfair than when they were fair, while the correct rejections in unfair lineups stayed at about the same rate. This aspect of the data is captured in the culprit-presence detection parameter *dP* in the 2-HT eyewitness identification model. Furthermore, the results of both reanalyses^48,59^ showed a decreased guessing-based selection among the lineup members (parameter *g*) in unfair compared to fair lineups. Even without using a formal model of the processes underlying eyewitness performance, the surface-level data already suggest that guessing-based selection among the lineup members occurred less frequently when the lineup was unfair than when it was fair: Participants in both studies produced fewer filler identifications when lineups were unfair than when they were fair. Guessing-based selection among lineup members may have been discouraged in unfair lineups due to the poor match of the fillers to the culprit.

The 2-HT eyewitness identification model is able to capture all of these changes in performance because its parameters are based on the full 3 × 2 data structure to distinguish between culprit-presence detection, culprit-absence detection, biased selection and guessing-based selection so that the model is able to capture changes in those data categories that are often ignored when lineup performance is analyzed. Our findings thus suggest that fair lineups produce better outcomes than unfair lineups because they decrease biased selection of the suspect and not because they improve culprit-presence detection [in line with the conclusions of^14,62^].

## Manipulations of guessing-based selection: Validation of parameter *g*

The next step was to test the validity of parameter *g,* which reflects the probability of selecting one of the lineup members based on guessing, a process that occurs alarmingly frequently not only in the laboratory but also in real-world lineups in which selecting a lineup member as the culprit may have serious consequences^63^. A straightforward and reliable way to manipulate guessing-based selection is to use instructions designed to manipulate the participants’ expectations about what they will encounter [e.g.,^34^]. In the context of lineups, so-called ‘biased’ pre-lineup instructions insinuate that the culprit is in the lineup and thus increase participants’ willingness to select one of the lineup members based on guessing when they are uncertain about whether or not the culprit is in the lineup^64–66^. The term ‘biased instructions’ is often used to refer to *one-sided* instructions that emphasize selectively the importance of selecting the culprit. The term ‘unbiased instructions’ is often used to refer to *two-sided* instructions that make participants aware of the fact that the culprit may or may not be in the lineup so that it is equally important to identify the culprit in culprit-present lineups and to reject culprit-absent lineups. Manipulating pre-lineup instructions therefore can be expected to affect the guessing-based selection parameter *g* and not the biased-suspect-selection parameter *b*. To avoid confusion, we therefore reserve the term ‘bias’ for the biased selection of the suspect in unfair lineups. We use the term one-sided instructions for instructions that emphasize selectively the need to identify the culprit and the term two-sided instructions for instructions that emphasize both the need to identify the culprit in culprit-present lineups and the need to reject culprit-absent lineups. One-sided instructions should encourage guessing-based selection, while two-sided instructions should discourage guessing-based selection. Here we reanalyzed datasets of Malpass and Devine ^67^ and of Lampinen et al. [^68^, Experiment 1] who had used one-sided and two-sided pre-lineup instructions.

### Effects of pre-lineup instructions on guessing-based selection: Reanalysis of Malpass and Devine^67^

Malpass and Devine^67^ influenced their participants’ guessing behavior by manipulating pre-lineup instructions that either insinuated or did not insinuate that the culprit was in the lineup. Malpass and Devine found that participants were more likely to choose one of the lineup members when one-sided instructions were given than when two-sided instructions were given, reflecting a higher prevalence of guessing-based selection after one-sided instructions. If parameter *g* of the 2-HT eyewitness identification model validly reflects guessing-based selection, then parameter *g* should be higher under one-sided than under two-sided instructions.

#### Method

Malpass and Devine^67^ randomly assigned 100 students to one of the four conditions of a 2 (lineup instruction: one-sided vs. two-sided) × 2 (culprit presence: present vs. absent) between-subjects design. The students witnessed a staged act of vandalism during a biofeedback demonstration at the university. They were exposed to a male confederate (visible for 85 s) who damaged the electrical equipment before he fled the room. On one of three evenings following the act of vandalism, participants viewed a simultaneous live lineup consisting of five persons who were lined up against the wall of a room. Half of the lineups included the culprit and four innocent fillers (culprit-present lineups), while the remaining lineups consisted of five innocent fillers (culprit-absent lineups). All fillers matched the appearance of the culprit. The position of each lineup member was counterbalanced. Before viewing the lineup, participants read either one-sided or two-sided printed lineup instructions. The one-sided instructions led participants to believe that the culprit was present. The students were instructed to choose one of five numbers (one number for each lineup member). There was no obvious option for rejecting the lineup. Instead, participants had to ask how to indicate such a response. In contrast, the two-sided instructions explicitly stated that the culprit may or may not be present and provided the participants with an option (circling number 0) to reject the lineup [for more details, see^67^].

#### Results

**Figure 4:**
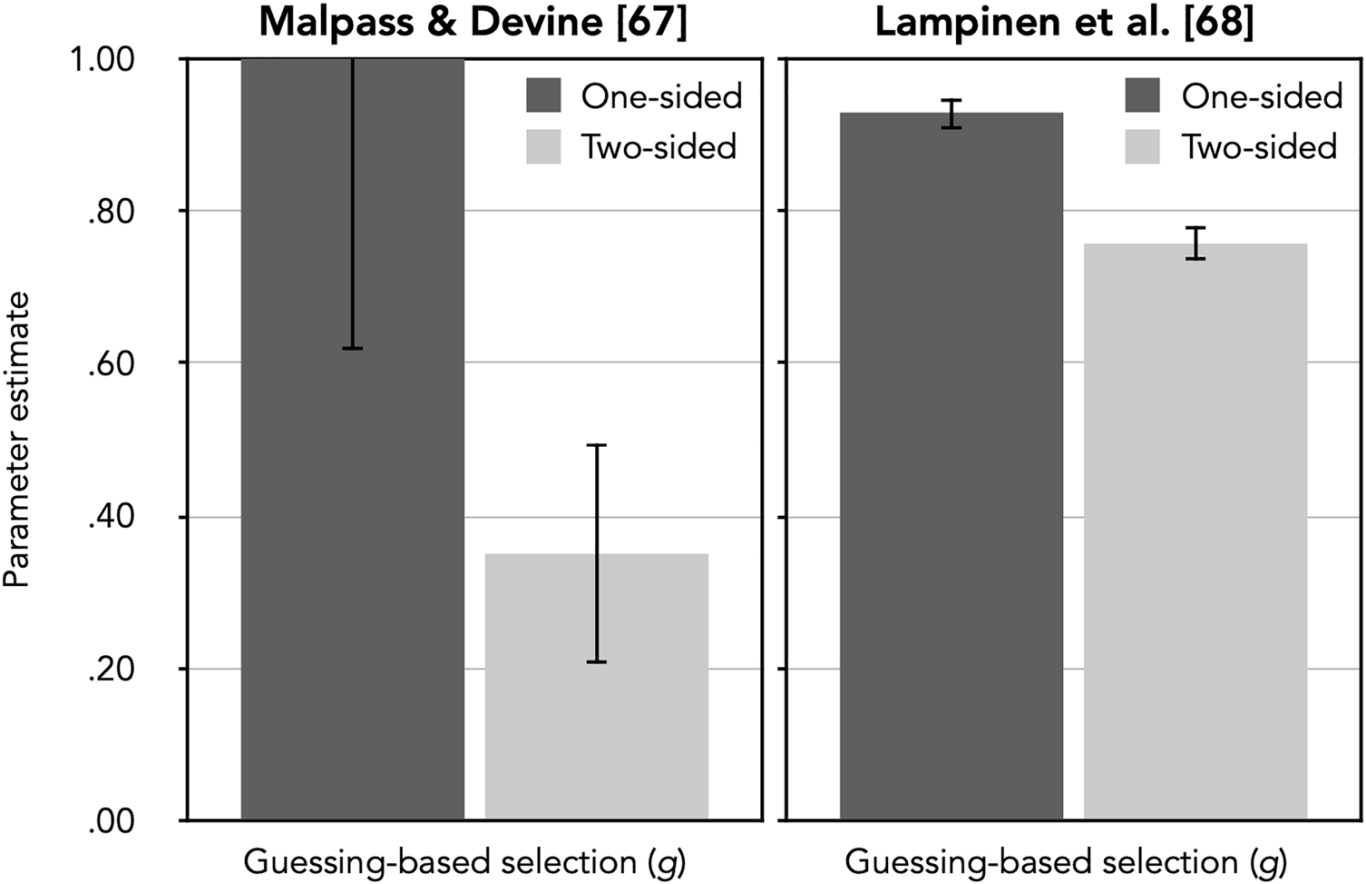
Estimates of parameter *g* (representing the probability of guessing-based selection among the lineup members) of the 2-HT eyewitness identification model when applied to the data reported by Malpass and Devine^67^ and by Lampinen et al. [^68^, Experiment 1] as a function of pre-lineup instructions (one-sided vs. two-sided). The error bars represent standard errors.

The observed response frequencies (see the upper half of Table 6) were reconstructed from the proportions reported by Malpass and Devine^67^ in their Table 1*. * Again, the number of innocent-suspect identifications was estimated by dividing the total number of false identifications in culprit-absent lineups by the number of lineup members [cf.^47,48^]. For the model-base d r e analysis, two sets of the trees shown in Fig. 1 were needed, one for the one-sided and one for the two-sided pre-lineup instructions. Parameters *dA* and *b* were each set to be equal between the conditions because there was no reason to assume that these parameters should differ as a function of the pre-lineup instructions. The model incorporating these restrictions was used as a compariso n standard for the subsequent nested likelihood-ratio tests, *G*^2^( 2) = 3.35, *p* = 0.187. The estimates of the guessing-based selection parameter *g* as a function of pre-lineup instructions are shown in the left panel of Fig. 4. Table 7 shows the estimates of parameters *dP*, *b* and *dA*.

**Table 6:** Response frequencies as reconstructed from Table 1 of Malpass and Devine [^67^, p. 485] and from Table 1 of Lampinen et al. [^68^, p. 412], see text for details.

	Culprit-present lineups			Culprit-absent lineups		
	Culprit identifications	Filler identifications	Lineup rejections	Innocent-suspect identifications	Filler identifications	Lineup rejections
**Malpass and Devine**^67^						
Pre-lineup instructions						
One-sided	21	7	0	3	14	5
Two-sided	19	0	4	2	7	18
**Lampinen et al.**^68^						
Pre-lineup instructions						
One-sided	68	88	10	25	126	15
Standard two-sided	60	85	22	19	97	50
Detailed two-sided	56	76	32	20	100	46

**Table 7:** Estimates of parameters *dP, b* and *dA* of the 2-HT eyewitness identification model for the data reported by Malpass and Devine^67^ and by Lampinen et al. [^68^, Experiment 1].

Malpass and Devine^67^				Lampinen et al.^68^			
Pre-lineup instructions	Parameter estimates			Pre-lineup instructions	Parameter estimates		
	*dP*	*b*	*dA*		*dP*	*b*	*dA*
One-sided	.68 (.11)	.01 (.06)	.21 (.30)	One-sided	.30 (.05)	.00 (.02)	.04 (.03)
Two-sided	.81 (.09)			Two-sided	.26 (.03)		

Values in parentheses represent standard errors. Within the model used as a comparison standard, parameter *dP* was estimated separately for one-sided and two-sided pre-lineup instructions. Parameters *b* and *dA* were each set to be equal between the one-sided and the two-sided pre-lineup instruction conditions. *dP* = probability of detecting the presence of the culprit; *b* = probability of biased suspect selection; *dA* = probability of detecting the absence of the culprit.

The guessing-based selection parameter was clearly affected by the manipulation of the pre-lineup instructions. Parameter *g* representing the tendency to select, in a state of uncertainty, one of the lineup members based on guessing was significantly higher under one-sided than under two-sided instructions, ∆*G*^2^(1) = 20.95, *p* < 0.001. By contrast, the culprit-presence detection parameter *dP* remained unaffected by the instructions, ∆*G*^2^(1) = 0.88, *p* = 0.34*7*.

### Effects of pre-lineup instructions on guessing-based selection: Reanalysis of Lampinen et al.^68^

Lampinen et al. [^68^, Experiment 1] manipulated participants’ guessing behavior in a similar fashion as Malpass and Devine^67^. While one-sided pre-lineup instructions were presented to encourage guessing-based selection, two versions of two-sided pre-lineup instructions were given to discourage guessing-based selection. The findings showed that the two-sided instructions significantly reduced inaccurate identifications^68^*.* Thus, in terms of the 2-HT eyewitness identification model, the guessing-based selection parameter *g* should be higher under one-sided instructions than under two-sided instructions.

#### Method

Lampinen et al. [^68^, Experiment 1] randomly assigned 995 students to one of the six experimental conditions resulting from a 3 (lineup instruction: one-sided vs. standard two-sided vs. detailed two-sided) × 2 (culprit presence: present vs. absent) between-subjects design. Participants viewed a 15-s video showing a woman stealing a backpack. After completing a 5-min distractor task, participants were given a paper copy of one of three types of instructions to read while the experimenter simultaneously read the instructions out loud. The one-sided instructions simply required the participants to identify the culprit in the lineup. The standard two-sided instructions contained the additional statement that the culprit may or may not be in the lineup. The detailed two-sided instructions were formulated according to the recommendations of major United States and international law enforcement agencies and were supplemented by statements that (a) it is just as important to clear an innocent person as it is to identify the culprit and (b) the police will continue to investigate the crime regardless of whether an identification is made or the lineup is rejected^69^. Participants were subsequently shown a simultaneous lineup consisting of six faces in a 3 × 2 array in which the culprit was either present or absent. Lampinen et al. created six versions of the culprit-present lineups by replacing one of the fillers with the culprit. All fillers matched the culprit’s description [for more details, see^68^, Experiment 1].

#### Results

The observed response frequencies (see the lower half of Table 6) were calculated from the proportions reported by Lampinen et al.^68^ in their Table 1. Because the exact number of participants for each condition was not provided, we assumed that participants were assigned in equal numbers to the conditions and divided the total number of participants by six (the number of conditions). The number of innocent-suspect identifications was estimated by dividing the total number of false identifications in culprit-absent lineups by the number of lineup members [cf.^47,48^]. Three sets of the trees shown in Fig. 1 were used, one for the one-sided instructions condition, one for the standard two-sided instructions condition and one for the detailed two-sided instructions condition. Parameters *dA* and *b* were each set to be equal among the conditions because there was no reason to assume that these parameters should differ as a function of the lineup instructions. For the sake of simplicity, the processing trees for the two types of two-sided instructions were combined by additionally assuming that guessing-based selection (*g*) and culprit-presence detection (*dP*) did not differ between more and less detailed two-sided instructions. This assumption was based on the results of Lampinen et al., who had found no differences in identification performance between the two types of two-sided lineup instructions. The model incorporating these restrictions was used as a comparison standard for the subsequent nested likelihood-ratio tests, *G*^2^(6) = 4.86, *p* = 0.562. The estimates of the guessing-based selection parameter *g* as a function of pre-lineup instructions are shown in the right panel of Fig. 4. Table 7 shows the estimates of parameters *dP*, *b* and *dA*.

The estimate of parameter *g,* which represents the probability of selecting, in a state of uncertainty, one of the lineup members based on guessing, was higher for one-sided instructions than for two-sided instructions. This difference was statistically significant, ∆*G*^2^(1) = 36.39, *p* < 0.001. By contrast, the culprit-presence detection parameter *dP* did not significantly differ between the types of instructions, ∆*G*^2^(1) = 0.69, *p* = 0.406.

### Discussion

The model-based results of both reanalyses^67,68^ showed that parameter *g*, representing the probability of selecting one of the lineup members based on guessing, were consistently higher for one-sided than for two-sided pre-lineup instructions. The fact that the guessing-based selection parameter sensitively reflected the experimental manipulations of the pre-lineup instructions in both reanalyses further supports the validity of the 2-HT eyewitness identification model.

## Manipulations of culprit-absence detection: Validation of parameter *dA*

The final step in the model validation presented here concerns parameter *dA,* which represents the probability of detecting the absence of the culprit. In order to validate this parameter, an experimental manipulation is needed that affects the proportion of correct rejections of culprit-absent lineups. To this end, Winter et al.^3^ constructed culprit-absent lineups in which all members had conspicuous birthmarks, which was not the case for the culprit. Given that none of the members in the culprit-absent lineups resembled the culprit, it was relatively easy to detect the absence of the culprit and thus to reject the culprit-absent lineups. As expected, parameter *dA* was significantly higher when the culprit-absent lineups were easy to reject. The obvious and downright trivial manipulation of the detection of culprit absence used by Winter et al. is ideal for the purpose of the model validation. However, it seems also interesting to explore whether there are factors that facilitate culprit-absence detection in more realistic settings. In fact, it would be highly desirable to find methods that actually improve the witnesses’ ability to detect the absence of the culprit and to specifically reject culprit-absent lineups, ideally without affecting guessing-based selection. Two such methods are reported below.

The first method is the use of a wildcard, that is, a silhouette with a question mark that represents a ‘mystery man’ that can be chosen instead of the suspect or one of the fillers. The wildcard option was introduced to make it easier for children to reject culprit-absent lineups by providing an option to reject the lineup that is more equivalent to a positive response when choosing the suspect^70^. In the majority of studies available to date, an intriguing pattern of results has emerged: A wildcard decreases the rate of false identifications in culprit-absent lineups without increasing the rate of false rejections of culprit-present lineups^71–73^. This pattern of results suggests that the effect of using a wildcard does not affect guessing-based selection—in which case it should have increased both correct *and* false lineup rejections—but may specifically improve the detection of the absence of the culprit.

The second method is the use of a culprit-absent practice lineup^74^ that has been introduced to facilitate the rejecting of culprit-absent lineups for older adults. Just as the wildcard, the culprit-absent practice lineup has led to an increase in correct culprit-absent lineup rejections without affecting the rate of false culprit-present lineup rejections, suggesting that the underlying process is a facilitation of the detection of the culprit absence and not a decrease in guessing-based selection.

If the 2-HT eyewitness identification model is valid, then the effects of wildcards^70^ and culprit-absent practice lineups^74^ should be reflected in parameter *dA,* which was designed to measure the detection of the absence of the culprit. We tested this assumption by reanalyzing the data obtained by Karageorge and Zajac^70^ and by Wilcock and Bull [^74^, Experiment 2].

### Effects of a wildcard on culprit-absence detection: Reanalysis of Karageorge and Zajac^70^

The results of a number of studies have shown that children appear to have considerable difficulty rejecting lineups even if the culprit is absent [for a review, see^75^]*.* Karageorge and Zajac^70^ aimed to enhance children’s ability to reject culprit-absent lineups by inserting a wildcard within the lineup that could be chosen instead of one of the lineup members. Children were more likely to reject the culprit-absent lineup when a wildcard was provided than when no such option was provided. Interestingly, the rate of correct rejections of culprit-absent lineups increased, whereas the rate of false rejections of culprit-present lineups did not. If a wildcard would simply affect the probability of selecting one of the lineup members based on guessing, it should have affected correct and false rejections equally. The selective effect of a wildcard on correct lineup rejections thus can only be caused by an increased detection of the absence of the culprit. In the 2-HT eyewitness identification model, a wildcard manipulation thus should selectively affect the culprit-absence detection parameter *dA*. Specifically, parameter *dA* should be higher in the wildcard condition than in the control condition.

#### Method

Karageorge and Zajac^70^ randomly assigned younger (age: 5 to 7 years, *n* = 101) and older (age: 8 to 11 years, *n* = 109) children to one of the eight conditions of a 2 (wildcard condition: wildcard vs. control) × 2 (culprit presence: present vs. absent) × 2 (delay: 1 to 2 days vs. 2 weeks) between-subjects design. During a visit to a fire station, the children were exposed for 30 to 45 s to a male confederate (henceforth referred to as the culprit) sliding down a fire pole. Either 1 to 2 days or 2 weeks after the event, all children who stated that they remembered the visit to the fire station (*n* = 204) were presented with a six-person culprit-present or culprit-absent lineup. The culprit-absent lineup contained the same five fillers as the culprit-present lineup, but the culprit was replaced by an innocent suspect who most resembled the culprit while the other fillers also shared basic characteristics with the culprit. The lineup photos were placed on a table in two rows of three. In the wildcard condition, a photo of a silhouette with a superimposed question mark was placed between the two rows. Prior to viewing the photos, all children were given standard two-sided pre-lineup instructions. Children in the control condition were instructed to point to the photo of the culprit if it was present and to tell the experimenter if it was not. Children in the wildcard condition were instructed to point to the photo of the culprit if it was present and to the silhouette (denoted as “this special photo”) if it was not [for more details, see^70^].

#### Results

**Figure 5:**
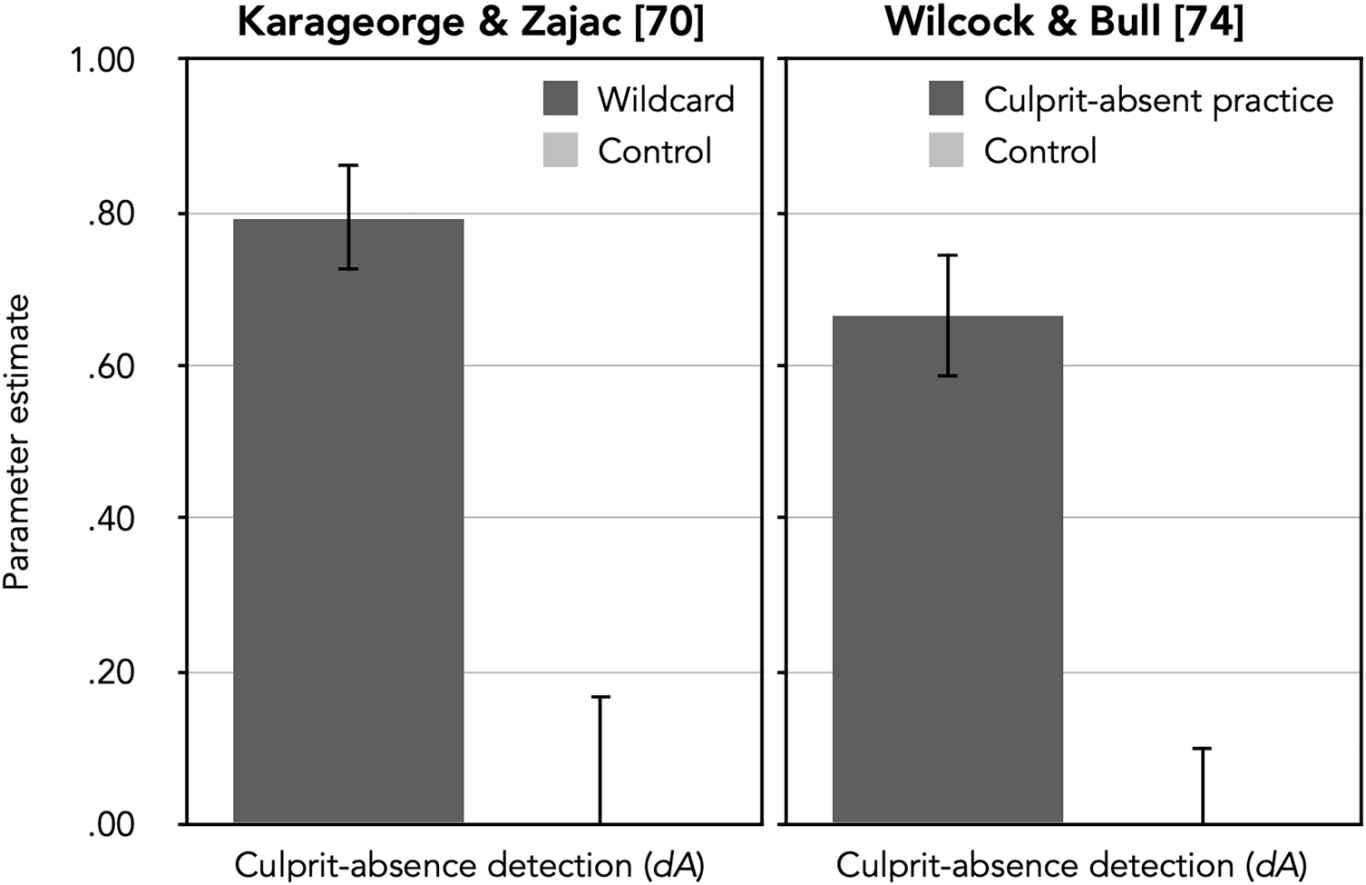
Estimates of parameter *dA* (representing the probability of detecting the absence of the culprit) of the 2-HT eyewitness identification model when applied to the data reported by Karageorge and Zajac^70^ and by Wilcock and Bull [^74^, Experiment 2] as a function of the wildcard condition (wildcard vs. control; left panel) and the pre-lineup procedure (culprit-absent practice vs. control; right panel). The error bars represent standard errors.

The observed response frequencies (see the upper half of Table 8) were reconstructed from the proportions reported by Karageorge and Zajac^70^ in their Table 1 which the data were already collapsed over the delay conditions (1 to 2 days vs. 2 weeks). Given that the total number of children in each age group in the final sample was not specified, it was not possible to analyze the data for younger and older children separately. Therefore, we collapsed the data across age groups. According to Karageorge and Zajac [^70^, p. 168], half of the children were presented with the culprit-present lineup, while the remaining children were presented with the culprit-absent lineup. More specifically, in the control condition, 53 of the 107 children saw the culprit-present lineup and 54 children saw the culprit-absent lineup, whereas in the wildcard condition, 48 of the 97 children saw the culprit-present lineup and 49 children saw the culprit-absent lineup. For the model-based reanalysis, we needed two sets of the model trees depicted in Fig. 1, one for the wildcard condition and one for the control condition. The *b* parameters were set to be equal between the conditions because there was no reason to assume that these parameters should differ as a function of the presence or absence of the wildcard. The model incorporating these restrictions was used as a comparison standard for the subsequent nested likelihood-ratio tests, *G*^2^(1) = 3.58, *p* = 0.059. The estimates of the culprit-absence detection parameter *dA* as a function of the wildcard condition are shown in the left panel of Fig. 5. Table 9 shows the estimates of parameters *dP*, *b* and *g*.

**Table 8:** Response frequencies as reconstructed from Table 1 of Karageorge and Zajac [^70^, p. 173] and from Table 3 of Wilcock and Bull [^74^, p. 730], see text for details.

	Culprit-present lineups			Culprit-absent lineups		
	Culprit identifications	Filler identifications	Lineup rejections	Innocent-suspect identifications	Filler identifications	Lineup rejections
**Karageorge and Zajac**^70^						
Wildcard condition						
Wildcard	31	10	7	2	6	41
Control	36	8	9	25	13	16
**Wilcock and Bull**^74^						
Pre-lineup procedure						
Practice	24	21	5	2	12	36
Control	20	20	10	7	36	7

**Table 9:** Estimates of parameters *dP, b* and *g* of the 2-HT eyewitness identification model for the data reported by Karageorge and Zajac^70^ and by Wilcock and Bull [^74^, Experiment 2].

Karageorge and Zajac^70^				Wilcock and Bull^74^			
Wildcard condition	Parameter estimates			Pre-lineup procedure	Parameter estimates		
	*dP*	*b*	*g*		*dP*	*b*	*g*
Wildcard	.37 (.15)	.37 (.09)	.67 (.10)	Practice	.40 (.09)	.00 (.05)	.83 (.07)
Control	.45 (.14)		.51 (.11)	Control	.31 (.09)		.80 (.06)

Values in parentheses represent standard errors. Within the model used as a comparison standard, parameters *dP* and *g* were estimated separately for the wildcard and the control conditions^70^ and for the culprit-absent practice and the control conditions [^74^, Experiment 2]. Parameter *b* was set to be equal between the experimental conditions. *dP* = probability of detecting the presence of the culprit; *b* = probability of biased suspect selection; *g* = probability of guessing-based selection among the lineup members.

The wildcard manipulation affected the culprit-absence detection parameter *dA* as expected. The probability of detecting the absence of the culprit was significantly higher when a wildcard was presented than when it was not, ∆*G*^2^(1) = 29.79, *p* < 0.001. The wildcard manipulation did not affect the probability of detecting the culprit as measured by parameter *dP,* ∆*G*^2^(1) = 0.21, *p* = 0.646. The guessing-based selection parameter *g* was not affected by the wildcard manipulation either, ∆*G*^2^(1) = 1.44, *p* = 0.229.

### Effects of a culprit-absent practice lineup on culprit-absence detection: Reanalysis of Wilcock and Bull^74^

Just like children, older adults are less likely to correctly reject a culprit-absent lineup than younger adults [for a meta-analysis, see^76^]. Wilcock and Bull [^74^, Experiment 2] examined the effect of a culprit-absent practice lineup on correct lineup rejections. Participants in the culprit-absent practice lineup condition correctly rejected culprit-absent lineups more often than participants in the control condition without a culprit-absent practice lineup. Interestingly, the culprit-absent practice lineup did not increase false rejections of culprit-present lineups. This pattern of results suggests that guessing-based selection cannot be responsible for the effect of the culprit-absent practice lineup because guessing-based selection would have decreased the rate of rejections of both culprit-present and culprit-absent lineups. Instead, the culprit-absent practice lineup must have improved the detection of the absence of the culprit because only this explanation is consistent with a selective increase in correct rejections of culprit-absent lineups. Therefore, it can be predicted that the culprit-absent practice manipulation should affect the culprit-absence detection parameter *dA.* Specifically*,* parameter *dA* should be significantly higher in the culprit-absent practice lineup condition than in the control condition.

#### Method

Wilcock and Bull [^74^, Experiment 2] randomly assigned 100 older participants to one of two groups (culprit-absent practice: culprit-absent practice lineup vs. control). Culprit presence (present vs. absent) was a within-subjects factor. Participants were shown a 110-s video of two men breaking into a house. After a 30-min retention interval, half of the participants were presented with a culprit-absent practice lineup consisting of six color pictures of famous women. The participants were asked to identify the Queen of England and were also informed that her face may or may not be present (there was no picture of the Queen). All participants correctly rejected the lineup. After rejecting the lineup, participants were again warned that not all police lineups include the culprit and that even the police can make mistakes. Participants were then given standard two-sided pre-lineup instructions before viewing the real lineups. Wilcock and Bull constructed a culprit-present and a culprit-absent lineup for each of the two culprits consisting of six faces in a 3 × 2 array. Participants were shown one culprit-present and one culprit-absent lineup (i.e., the first participant saw a culprit-present lineup for culprit 1, followed by a culprit-absent lineup for culprit 2, the second participant saw a culprit-absent lineup for culprit 1 and a culprit-present lineup for culprit 2 and so on). For the sake of simplicity, we did not distinguish between the two culprits but aggregated the data. All fillers matched the culprits’ descriptions. In the culprit-absent lineups, the culprit was replaced by the filler who was rated as most similar-looking to the culprit. The culprit and the culprit replacement were randomly placed across all six lineup positions [for more details, see^74,^, Experiment 2].

#### Results

The observed response frequencies (see the lower half of Table 8) were taken from Table 3 of Wilcock and Bull^74^*.* As in the previous reanalyses, we estimated the number of innocent-suspect identifications using the standard procedure of dividing the total number of false identifications in culprit-absent lineups by the number of lineup members^47,48^. Two sets of the model trees depicted in Fig. 1 were needed, one for the culprit-absent practice lineup condition and one for the control condition. The *b* parameters were set to be equal between the conditions because there was no reason to assume that these parameters should differ as a function of the culprit-absent practice manipulation. The model incorporating these restrictions was used as a comparison standard for the subsequent nested likelihood-ratio tests, *G*^2^(1) = 2.92, *p* = 0.087. The estimates of culprit-absence detection parameter *dA* as a function of the culprit-absent practice manipulation are shown in the right panel of Fig. 5. Table 9 shows the estimates of parameters *dP*, *b* and *g*.

The culprit-absence detection parameter *dA* was significantly higher in the culprit-absent practice lineup condition than in the control condition, ∆*G*^2^(1) = 23.42, *p* < 0.001. By contrast, the detection of the culprit presence reflected in parameter *dP* remained unaffected by the culprit-absent practice manipulation, ∆*G*^2^(1) = 0.58, *p* = 0.444. The same was true for the guessing-based selection parameter *g*, ∆*G*^2^(1) = 0.18, *p* = 0.673.

### Discussion

The two final reanalyses demonstrated that manipulations designed to facilitate culprit-absence detection selectively affected parameter *dA* of the 2-HT eyewitness identification model. Karageorge and Zajac^70^ inserted a wildcard within the lineup. Wilcock and Bull [^74^, Experiment 2] presented a culprit-absent practice lineup. Both procedures selectively increased the rate of correct rejections of culprit-absent lineups but did not affect the rate of false rejections of culprit-present lineups, suggesting that introducing these procedures did not induce decreased guessing-based selection. The latter conclusion is consistent with the fact that guessing-based selection parameter *g* was not affected by either the wildcard or the culprit-absent practice lineup.

## General discussion

Here, we report a validation of the novel 2-HT eyewitness identification model using published data. The model simultaneously takes into account all of the data categories that are observed in lineups, that is, correct culprit identifications, false innocent-suspect identifications, false filler identifications in culprit-present and culprit-absent lineups, false rejections of culprit-present lineups and correct rejections of culprit-absent lineups. Based on these data, the model yields measures of latent processes that underlie eyewitness identification performance. Specifically, the model is designed to distinguish between two types of detection processes—detection of the presence of the culprit and detection of the absence of the culprit—as well as two different types of non-detection-based decision processes—biased selection of a suspect that stands out from the fillers in unfair lineups and selecting a lineup member based on guessing. We hope that distinguishing between these qualitatively different latent processes helps to improve the clarity of the interpretation of lineup data. A typical approach is to try to infer the underlying processes indirectly from surface-level data by comparing the rate of correct culprit identifications and false innocent-suspect identifications between different conditions, but often the problem arises that the same manipulation can simultaneously affect qualitatively different processes such as, for example, both culprit-presence detection and guessing-based selection^8,13^. The 2-HT eyewitness identification model serves to disentangle the effects of manipulations on the processes underlying eyewitness identifications by yielding separate measures of these latent processes and by enabling researchers to test hypotheses directly at the level of the parameters representing these latent processes. In that way, the model can be used to tackle important research questions such as, for example, the question of whether the process of culprit-presence and culprit-absence detection, biased selection and guessing-based selection differ between simultaneous and sequential lineups. However, before a measurement model such as the 2-HT eyewitness identification model can be used to tackle new and unresolved empirical questions, it is important to demonstrate that the model parameters sensitively reflect the processes they are intended to measure^19,29^.

In a separate empirical contribution, Winter et al.^3^ have reported fresh experiments that were designed with the sole purpose of testing whether the parameters of the 2-HT eyewitness identification model sensitively reflect specific manipulations that were carefully crafted to affect the latent processes underlying eyewitness identifications postulated by the model; these tests were successful. The analyses reported here complement the results reported by Winter et al. by demonstrating that the model parameters also sensitively reflect manipulations of the processes they were designed to measure in published studies obtained with a wide variety of different experimental manipulations, samples, paradigms and laboratory as well as real-world settings. First, it was demonstrated that parameter *dP* reacts sensitively to manipulations of encoding conditions that can be expected to affect the detection of the presence of the culprit. Specifically, the culprit-presence detection parameter *dP* was higher in response to long as opposed to short culprit exposure and under good as opposed to poor viewing conditions at encoding. Second, manipulations of lineup fairness affected the estimate of parameter *b*, designed to reflect the process of the biased selection of a suspect who stands out from the fillers. As expected under the assumption that this model parameter is valid, parameter *b* was higher for lineups with low as opposed to high suspect-filler similarity and for lineups in which the suspects had unique facial features distinguishing them from the fillers as opposed to lineups in which these distinctive features were concealed in all photos or replicated in the photos of the fillers. Third, manipulations of pre-lineup instructions had the predicted effects on the parameter representing the selection of a lineup member based on guessing. Parameter *g* was consistently higher when one-sided than when two-sided instructions were used. One-sided instructions implicate that the culprit is in the lineup, whereas two-sided instructions emphasize that the culprit may be present or absent. It should be uncontroversial that difficult encoding conditions should affect the process of culprit-presence detection, that biased suspect selection should be increased in unfair lineups and that participants can be discouraged to select a lineup member based on guessing by two-sided lineup instructions. Selecting manipulations that obviously affect the detection of the absence of the culprit is somewhat more difficult. Winter et al. have provided evidence showing that the detection of culprit absence is enhanced when all of the lineup members in the culprit-absent lineup can be easily ruled out based on salient perceptual features. Such a manipulation is unlikely to be found in the literature. This is so because such an experiment makes sense only in the context of model validation in which the ideal manipulation is obvious and trivial in the sense that there is broad agreement on the manipulation’s effect on certain cognitive processes. Therefore, our reanalysis-based test of the validity of the culprit-absence detection parameter concentrated on manipulations that were specifically designed to help children and older adults to reject culprit-absent lineups^70,74^. In previous studies these manipulations led to an increase in correct rejections of culprit-absent lineups while the false rejections of culprit-present lineups remained unaffected, suggesting that the affected process was the detection of the absence of the culprit and not the selection of a lineup member based on guessing. The present reanalysis confirms this conclusion and thereby provides further evidence for the process of culprit-absence detection: The use of a wildcard procedure and a culprit-absent practice lineup increased the estimates of culprit-absence detection parameter *dA* compared to standard lineup procedures.

The 2-HT eyewitness identification model distinguishes between two non-detection-based judgements: a process of selecting a lineup member based on guessing (parameter *g*) and a process of biased selection of a suspect who stands out from the fillers (parameter *b*). Traditionally, eyewitness researchers have relied on the mock-witness task to determine the degree to which a lineup is unfair. This task involves presenting a lineup and a description of a culprit to participants who did not witness the crime—so called mock witnesses—and then asking the mock witnesses to identify the person who best matches the description^77^. Based on the answers of the mock witnesses, various lineup fairness measures can be calculated, reflecting either the lineup size (i.e., how many lineup members have plausibility as the culprit?) or biased selection of the suspect (i.e., to what extent does the suspect stand out from other lineup members?^78,79^). However, some researchers have cautioned that these measures may suffer from low validity and reliability and have suggested to consider alternative methods for estimating lineup fairness^80^. The 2-HT eyewitness identification model offers such an alternative method for measuring and testing hypotheses about biased suspect selection. In fact, the 2-HT eyewitness identification model measures lineup fairness directly from the witnesses’ identification data—without relying on a separate paradigm involving mock witnesses. Based on this direct measurement, one can draw conclusions about the cognitive processes that determine the decisions of eyewitnesses that may, to some degree, differ from those of mock witnesses^81^.

The model also makes it possible to distinguish between two detection processes: the detection of the presence of the culprit (parameter *dP*) and the detection of the absence of the culprit (parameter *dA*). Traditional measures of lineup performance often provide only a single accuracy index that simultaneously accounts for the witness’s performance in culprit-present and culprit-absent lineups. However, it is possible to argue that detecting the presence of a culprit might be achieved by a different underlying process than detecting the absence of a culprit. The first piece of evidence supporting this argument is that the process of detecting the presence of the culprit varies as a function of manipulations that leave the process of detecting the absence of the culprit unaffected (see Fig. 2 and Table 3 above) and vice versa (see Fig. 5 and Table 9 above). The second piece of evidence is that in most of the data sets presented here, the probability of detecting the presence of the culprit was quite high, while the probability of detecting the absence of the culprit was considerably lower (the statistical test of a difference between *dP* and *dA* results in *p* < 0.05 for all reanalyses reported in the present article). Interestingly, it seems plausible that techniques developed to help children and older adults to reject culprit-absent lineups specifically affect the process of culprit-absence detection. However, without the help of a model-based analysis, Karageorge and Zajac^70^ and Wilcock and Bull^74^ had to rely on the observation that a wildcard procedure or a culprit-absent lineup practice selectively increased correct rejections of culprit-absent lineups with no effect on false rejections of culprit-present lineups, which indirectly suggests that these procedures may help to detect culprit absence and do not decrease guessing-based selection. With the 2-HT eyewitness identification model it is less indirect and more straightforward to conclude that these procedures enhance the detection of the culprit absence (parameter *dA*) while leaving guessing-based selection (parameter *g*) unaffected. We hope that by including a separate parameter for culprit-absence detection, the 2-HT eyewitness identification model will stimulate more research on techniques that specifically improve witnesses’ ability to detect the absence of a culprit, which seems highly desirable.

A limitation of the present reanalyses is that many studies in the eyewitness literature used only fillers in culprit-absent lineups [e.g.,^45,46,67,68,74^]. However, the model-based analyses make use of the full 2 × 3 data structure, which includes innocent-suspect identifications. To be able to perform a model-based analysis of experiments without a designated innocent suspect in culprit-absent lineups, we followed the standard procedure for estimating the rate of innocent-suspect identifications from the filler identifications by dividing the total number of false identifications in culprit-absent lineups by the number of lineup members^47,48^. This method rests on the assumption that the culprit-absent lineups contained an innocent suspect that was selected with the same probability as each of the fillers, which implies that the lineups were fair. Importantly, this assumption does not seem to affect the major conclusions that can be drawn from the data as the present reanalysis is consistent with the experimental validation study by Winter et al.^3^ in which we used designated innocent suspects in culprit-absent lineups. However, to validly measure lineup fairness and to increase the ecological validity of the analysis, we encourage researchers to include a designated innocent suspect in culprit-absent lineups in future studies. In the real world, the photographs of the suspects whose guilt or innocence is unknown to the police are often taken from different sources (e.g., social media) than the photographs of the fillers (e.g., a database). Photographs from different sources may differ systematically in certain characteristics. It thus greatly improves the ecological validity of the results to have a designated innocent suspect whose photograph deviates from the photographs of the fillers in the culprit-absent lineups in the same way as the photograph of the culprit deviates from the photographs of the fillers in the culprit-present lineups [cf.^3^].

Due to their mathematical and conceptual simplicity, the class of MPT models is ideally suited to develop simple formal measurement models to assess latent processes involved in specific empirical paradigms^18,19,29^. However, a more controversial property of MPT models is that they involve a threshold concept, thus assuming that recognition judgements result from discrete memory states rather than continuously distributed signal strength (as the SDT assumes). While some researchers argued that the threshold assumption is inconsistent with the available empirical evidence^82,83^, others have shown that threshold models and SDT-based models can both account for recognition memory performance^37,84,85^. The theoretical and practical usefulness of both approaches in the field of eyewitness identifications has to be further investigated in future research.

Given the well-documented evidence illustrating the fallibility of eyewitness testimonies, it remains an important goal to advance our knowledge about the latent processes underlying eyewitness identification decisions. We hope to contribute to this advancement by presenting a multinomial model for analyzing lineup performance, the 2-HT eyewitness identification model. By incorporating the entire 2 × 3 data structure of responses in lineup identification tasks, the model enables inferences about latent cognitive processes that are not accessible when standard measures of eyewitness identification accuracy are used. Here, we tested the validity of the model by applying it to published data. A series of eight reanalyses provides evidence in support of a successful model validation. Validations of culprit-presence detection (*dP*), biased suspect selection (*b*), guessing-based selection among the lineup members (*g*) and culprit-absence detection (*dA*) showed that the parameters sensitively reflected experimental manipulations of the processes they were designed to measure. We conclude that the 2-HT eyewitness identification model is promising and can complement existing tools to analyze eyewitness identifications in lineups.

## Data Availability

The multiTree equations and data files of all reanalyses are available in the OSF repository, https://osf.io/pjc5b.
